# Primary Care Providers’ Perspectives on Online Weight-Loss Programs: A Big Wish List

**DOI:** 10.2196/jmir.1955

**Published:** 2012-01-19

**Authors:** Kevin O Hwang, Heather L Stuckey, Monica C Chen, Jennifer L Kraschnewski, Samuel N Forjuoh, Jennifer M Poger, Kathleen M McTigue, Christopher N Sciamanna

**Affiliations:** ^1^Department of Internal MedicineThe University of Texas Medical School at HoustonHouston, TXUnited States; ^2^Department of MedicinePenn State College of MedicineHershey, PAUnited States; ^3^Department of Family & Community MedicineTexas A&M Health Science Center College of MedicineScott & White HealthcareTemple, TXUnited States; ^4^Department of MedicineUniversity of PittsburghPittsburgh, PAUnited States

**Keywords:** Obesity, weight management, primary care

## Abstract

**Background:**

Integrating online weight-loss programs into the primary care setting could yield substantial public health benefit. Little is known about primary care providers’ perspectives on online weight-loss programs.

**Objective:**

To assess primary care providers’ perspectives on online weight-loss programs.

**Methods:**

We conducted focus group discussions with providers in family medicine, internal medicine, and combined internal medicine/pediatrics in Texas and Pennsylvania, USA. Open-ended questions addressed their experience with and attitudes toward online weight-loss programs; useful characteristics of existing online weight-loss programs; barriers to referring patients to online weight-loss programs; and preferred characteristics of an ideal online weight-loss program. Transcripts were analyzed with the grounded theory approach to identify major themes.

**Results:**

A total of 44 primary care providers participated in 9 focus groups. The mean age was 45 (SD 9) years. Providers had limited experience with structured online weight-loss programs and were uncertain about their safety and efficacy. They thought motivated, younger patients would be more likely than others to respond to an online weight-loss program. According to primary care providers, an ideal online weight-loss program would provide—at no cost to the patient—a structured curriculum addressing motivation, psychological issues, and problem solving; tools for tracking diet, exercise, and weight loss; and peer support monitored by experts. Primary care providers were interested in receiving reports about patients from the online weight-loss programs, but were concerned about the time required to review and act on the reports.

**Conclusions:**

Primary care providers have high expectations for how online weight-loss programs should deliver services to patients and fit into the clinical workflow. Efforts to integrate online weight-loss programs into the primary care setting should address efficacy and safety of online weight-loss programs in clinic-based populations; acceptable methods of sending reports to primary care providers about their patients’ progress; and elimination or reduction of costs to patients.

## Introduction

One-third of US adults are obese and another third are overweight [1]. The US Preventive Services Task Force recommends that clinicians provide or refer obese adults to high-intensity weight-loss counseling, defined as more than one session per month for at least the first 3 months [2]. Given that many primary care providers lack the time, skills, and supportive infrastructure to provide this level of counseling [3-5], there is an urgent need to identify effective weight-loss resources to which primary care providers can refer their patients.

Online weight-loss programs, with their interactive capabilities and wide reach, have been recognized as potential alternatives to traditional weight-loss programs [6-12]. Establishing partnerships between primary care providers and effective online weight-loss programs could create a substantial public health benefit in which partners play complementary roles to offer the patient a convenient and comprehensive weight-loss service. For example, the primary care provider could identify patients who need to and desire to lose weight, conduct a medical evaluation, and refer eligible patients to the online weight-loss program. Depending on available resources, the online weight-loss program could provide structured counseling, nutrition and exercise monitoring with feedback, and social support [13-16].

Prior studies have assessed patient perspectives on a primary care provider–online weight-loss program partnership [17] as well as clinicians’ perspectives on referring patients to weight-loss and diabetes self-education resources [18,19]. Integrating online weight-loss programs into routine primary care will require a thorough understanding of primary care providers’ perspectives [5], but such knowledge is lacking. Therefore, a grounded theory approach was used to examine possible theoretical explanations for primary care providers’ experiences, attitudes, and preferences with respect to partnerships between primary care providers and online weight-loss programs in routine clinical care.

## Methods

### Recruitment

We conducted 9 focus group discussions with primary care providers from southeast Texas, central Texas, and central Pennsylvania (Table 1).

Providers were eligible if they practiced general internal medicine or family medicine in the outpatient setting. Participants included physicians, nurse practitioners, and physician assistants. A coordinator at each institution invited potentially eligible primary care providers by email and/or phone and confirmed eligibility.

**Table 1:** Characteristics of primary care practices for 9 focus group discussions

		Site A				Site B	Site C
Number of focus groups		3				2	4
Practice setting		Urban, academic				Urban, community-based, affiliated with medical school	Suburban, academic
Location		Southeast Texas				Central Texas	Central Pennsylvania
Prevalence of obesity		36.7%^a^				26.1%–31.4%^b^	31.2%^c^
		General internal medicine clinic 1	General internal medicine clinic 2	Family medicine clinic 1	Family medicine clinic 2		
Payer							
	Managed care	54%	66%	65%	1%	62%	50%
	Medicaid	9%	4%	5%	6%	6%	10%
	Medicare	36%	27%	20%	9%	30%	37%
	Self-pay, uninsured, or other	1%	2%	11%	84%	2%	3%

^a^ Prevalence of obesity among adults (age ≥18 years) seen at general internal medicine and family medicine clinics in 2009–2010. Source: electronic medical records.

^b^ Prevalence of obesity among adults (age ≥20 years) in counties served by site B, 2008. Source: Centers for Disease Control and Prevention, National Diabetes Surveillance System.

^c^ Prevalence of obesity among adults (age ≥20 years) in county served by site C, 2008. Source: Centers for Disease Control and Prevention, National Diabetes Surveillance System.

### Data Collection

The focus groups were facilitated by a general internist with public health training and experience with focus group discussions (KH), a medical student who observed and assisted in leading groups before leading a group (MC), a doctorate-level educator with experience with focus group discussions (HS), and a master’s-level educator who observed and assisted in leading groups before leading a group (JP). At the beginning of each session, participants completed an informed consent form as well as demographic and practice characteristic questionnaires. The facilitators asked open-ended questions to begin the session, using a semistructured standard interview script based on the research objectives. Questions addressed obesity in adults rather than children or adolescents.

The discussions began with introductory questions about how the primary care providers attempted to help their patients lose weight. The current analysis focused on questions related to primary care providers’ experience with and attitudes toward online weight-loss programs; useful characteristics of existing online weight-loss programs; barriers to referring patients to online weight-loss programs; and preferred characteristics of an ideal online weight-loss program (Textbox 1). The discussions were recorded and transcribed verbatim. Each focus group lasted approximately 45–60 minutes, and participants received a US $100 gift certificate.

**Focus Group Questions Related to Online Weight-Loss Programs**

- If you refer patients to online weight-loss programs, which programs do you use?
- If you had an online and/or in-person resource that you could offer in your clinic to help your patients lose weight that required a minimal time commitment from staff, how interested would your clinic be?
- What characteristics of online weight-loss programs have you found useful for patients?
- What are the top reasons why you don’t refer patients to an online weight-loss program?
- What could be offered in an online program that would make you want to refer patients to it?

### Data Analysis

The main sources of data were the focus group transcripts, but field notes also included reflections about the focus groups, the settings and culture of the sites, and nonverbal cues during the discussions. Because we found no prior scientific literature on primary care providers’ perspectives on online weight-loss programs, there was no well-defined theoretical framework to inform data analysis. Therefore, we used grounded theory to guide the analysis of data [20]. In the grounded theory approach, theory is developed from the data. Features of grounded theory are (1) use of a theoretical sample, (2) constant comparison of data against theoretical categories, and (3) focus on the development of theory via thematic saturation of categories [21]. Three investigators (MC, JK, KH) reviewed the transcripts and field notes, using manual open coding to identify categories, and 3 investigators (JK, KH, HS) convened to discuss common themes within the categories, and compared emergent data against the categories. Disagreements were resolved by consensus. After the ninth focus group, we had the opportunity to conduct an additional group, but we determined that thematic saturation was reached. The study was approved by the institutional review boards of all three sites.

## Results

The 9 focus groups included 44 primary care providers with mean age of 45 (SD 9) years (Table 2).

Three major themes from the focus groups were related to barriers to referring patients to online weight-loss programs, while an additional theme identified the characteristics and features of an ideal online weight-loss program (Textbox 2).

**Table 2:** Characteristics of focus group participants (N = 44)

Characteristic		n	%
Gender, male		25	57
**Specialty**			
	Family medicine	29	66
	Internal medicine	12	27
	Internal medicine and pediatrics	3	7
**Level**			
	Physician	40	91
	Nurse practitioner or physician assistant	4	9
Ethnicity, Hispanic		3	7
**Race**			
	White	32	73
	Black	1	2
	Asian	10	23
	Missing data	1	2

**Major themes from focus group discussions with primary care providers about their perspectives on online weight-loss programs**

1. Unfamiliar with online weight-loss programs
2. Uncertain about safety and efficacy of online weight-loss programs
3. Online weight-loss program appropriate only for motivated, technically savvy patients
4. Characteristics and features on an ideal online weight-loss program
  - Free
  - Structured curriculum
  - Goal-setting assistance
  - Self-monitoring tools
  - Psychologically oriented content
  - Peer support
  - Reports for primary care providers

### Primary Care Providers Unfamiliar With Online Weight-Loss Programs

Primary care providers generally reported that they referred their patients to structured weight-loss programs (such as Weight Watchers) or specialists, or they provided counseling within the clinical setting themselves. Many primary care providers had not referred their patients to online weight-loss programs because they were not familiar with them. One said that primary care providers were not educated about online weight-loss programs and that he didn’t know of any “online resources to help my patients out with obesity or help them lose weight,” and another stated “I’m not aware of them.” One participant said:

*It’s not something that I’ve routinely done to make specific referrals to online sites and I think as others have said, maybe it’s something where I’m just not very knowledgeable of what is available for both professionals and patients.*

Some primary care providers had heard of educational websites with obesity-related information, such as MyPyramid.gov, Diabetes.org, and WebMD.com, but were not familiar with “a weight-loss program per se.” Others were aware of formal online weight-loss programs such as WeightWatchers.com, SparkPeople.com, CalorieKing.com, FitDay.com, and MyFitnessPal.com, but few had referred patients to them.

### Uncertainty About Safety and Efficacy of Online Weight-Loss Programs

Even without being aware of existing online weight-loss programs, the primary care providers expressed uncertainty about the safety and efficacy of online weight-loss programs in general. The main safety concern was that online weight-loss programs would sell unsafe or untested weight-loss medications, either directly or via third-party advertisements. One said, “You’ll have a lot of people selling you products that contain unknown chemicals in them and you may make the situation worse.”

I’ve had patients come with websites for me to look at where they’re being sold something, a stimulant, cathartics, what have you. I think that whatever I’m going to recommend to a patient I have to have gone to myself and look at it. If there’s a website where somebody is selling something, that’s just not one I would recommend.

But that’s the biggest concern I have too, that even on a decent site, they are going to be funded. Whoever is funding them is going to set up their little advertisements too. It might be sending the wrong message at times.

The primary care providers did not specify a degree of weight loss (eg, produce 5% or 10% weight loss) attributed to participation in an online weight-loss program that would increase the likelihood of referring their patients. Acknowledging the paucity of evidence in support of common primary care interventions, a few primary care providers were hesitant to refer patients to online weight-loss programs without evidence of efficacy. One said “I haven’t looked at these carefully enough to know...this is one I like and there’s good evidence that it works, and I endorse it and suggest you use it.”

I guess with a lot of the therapeutics that we apply or recommend as physicians, sometimes the evidence base is not so strong and I think we all realize that. So, certainly I guess that if we thought that it wasn’t helpful, I guess we might be less inclined to recommend.

### Online Weight-Loss Program Appropriate Only for Motivated, Technically Savvy Patients

Primary care providers thought the online weight-loss programs would be most appropriate for patients who already had skills and self-motivation to lose weight. One provider thought that referring patients to an online weight-loss program would have limited impact because “so many of the people aren’t ready to lose weight” and “they just can’t get themselves motivated to do so.” Another participant thought that online weight-loss programs could be effective when used “in the right place by the right people...in the right frame of mind.”

Some people need handholding—they really want personal interaction. And then there seems to be a group that are self-starters, that are disciplined—that they keep track on their iPhone or program what they eat...They’re able to, on their own, make adjustments. The online thing seems to fall kind of in the middle.

If it’s online, it could be hit or miss and you would have to have a highly motivated person to keep coming back.

They also thought that older, poorer, or less-educated patients would not or could not access the online weight-loss programs. One provider said “I do have some patients that don’t have consistent access or a computer,” while another said:

A lot of my patients are Medicaid patients and I don’t think they have computer access to begin with. And if they do have computer access, they’re using it for recreational purposes.

In summary, most primary care providers were not familiar with online weight-loss programs and they expressed concerns about safety and efficacy. They typically believed online weight-loss programs were most suitable for highly motivated patients who were comfortable with using computers and the Internet.

### Characteristics and Features of an Ideal Online Weight-Loss Program

None of the 44 primary care providers claimed to have found an online weight-loss program with all the critical elements that they thought would help patients lose weight. We asked participants to envision the characteristics of a hypothetical, ideal online weight-loss program. The major findings are presented below.

#### Free

Primary care providers emphasized the importance of patients accessing an online weight-loss program for free. They said they would be more likely to refer patients and that patients would be more likely to join an online weight-loss program that was free.

#### Structured Curriculum

Primary care providers favored a structured behavioral program with a scheduled curriculum instead of a collection of self-directed resources. Without structure, an online resource would be just like a book: “I don’t think that’s terribly effective.”

It’s informal but it’s a structured program, allows them to record their caloric intake and caloric expenditure, and gives them some limits that they need to work within depending on what their weight-loss goals are so it’s been a nice tool to recommend to people.

If you had a way to generate reminders to people that are visiting the site to say, “hey, did you meet your weight-loss goal this week?,” or some type of system so that they don’t always have to self‐initiate...People don’t want to have a flooded amount of messages from this online weight-loss resource, but it would be kind of nice to know that they’re getting reminded...

#### Goal-Setting Assistance

According to primary care providers, an online weight-loss program should help patients define personal goals. It was also deemed important that users be able to specify “which barrier they want to tackle and how they might choose to do that.”

Something that matches the patient’s goals, I think, is what’s going to be the key. If it’s just a series of things that they can do and they’re not buying into any [of] them, I don’t think they’ll be successful. So, I think the motivational part of it has to be what can you see yourself doing moving forward.

They can set a goal weight, so they have a goal that they’re shooting for. Then, it interacts with them and gives them a number of calories that they can consume during the day and also then if they exercise, it adds that into the mix.

#### Self-monitoring Tools

Another feature valued by primary care providers was self-monitoring tools for diet, exercise, and weight. They recognized the opportunity for online weight-loss programs to facilitate the process of self-monitoring of food intake by automatically calculating the calorie content of foods. They felt that the burden of manually entering calories was too high for patients.

[Patients] want something that will kind of show them what they’re doing, something that makes it a little bit easier to count their calories.

I’m a firm believer in you got to do a food diary...The way to do it needs to be easy. There needs to be no calculation. There needs to be no nothing. So, like to drink a soda, there’s a drop‐down list...It’s gotta be easy. Not even saying the calories in it, just let it calculate the calories and give you some analysis later. People need to do no analysis. They just need to report.

#### Psychologically Oriented Content

Primary care providers felt that an online weight-loss program should offer more than information, that it should also address other mental processes crucial to a successful weight-loss effort. For example, one participant thought it was important that an online weight-loss program address “the motivational aspect of it, and also the implementation...some decision‐making and cognitive informational piece to it.” Another provider suggested a problem-solving component: “So, if they don’t meet their goal for the week, why did it go wrong? How are they going to get it back on track?” Even straightforward feedback on weight status could be accompanied by psychologically oriented content, such as the following:

...motivation, encouragement, and clearly showing results and benefits to why it’s helping you, like showing like in graphs what weight you’ve lost, how your [body mass index] is changing, how this minimizes your risk factors for heart disease and blood pressure...

#### Peer Support

Primary care providers recognized the potential value of peer support among users of an online weight-loss program in providing accountability as well as a venue for discussing sensitive issues in “a semi-anonymous fashion.” They thought that online peer support could mimic the support from typical group settings. (“Some people would respond to a group setting and so you can obviously do that online.”) Connecting patients to other individuals who shared the same struggles would also differentiate an online weight-loss program from less-interactive weight-loss resources, such as books.

I think the most important thing is relationships and talking with people, or being accountable to another human being and relating one on one. To the extent that an online program can either simulate a human interaction, or make use of actual people and their experiences, and facilitate experiences through technology, then I think that is an important part. Otherwise, it’s like reading a book.

But the primary care providers were also concerned that online support venues would be a source of “a bunch of bad advice” or “ideas being promulgated as official stuff that’s not really correct.” One solution would be to have the peer forums monitored by experts.

I like having a refereed group where you’ve got somebody with some education that’s chiming in periodically. It’s like a group visit in your office, where you’ve got someone that’s educated in that area guiding the group so that if they get off track that you can bring them back. Otherwise I would think that an online discussion would quickly turn into the latest fad.

#### Reports for Primary Care Providers

Some primary care providers would welcome reports from the online weight-loss program about their patients’ progress or the ability to “check in and see if the patient was using it.” The primary care providers anticipated using the reports as a framework for providing praise, support, and accountability either during or between office visits. The reports would position the primary care providers as an accountability partner in the patient’s weight-loss effort, because the patient would know “that stuff’s going to be going to the physician for review too.” The patient would “know you are watching them, instead of them just going off to a website somewhere.” This knowledge about the provider’s involvement was seen as a motivational factor for patients.

I would like to get information. I think if you get it at some kind of pattern, if you’re able to respond back to the patient, it makes them accountable, and they might be a little more motivated...They start to worry about their weight, you know, just a few days before the visit, but if they know you’re getting things all the time I think it might just be a little more motivation for them.

I think to have the actual information available would be a good thing for those people you can call and congratulate or just have a nursing staff just say, “Hey looks like you made progress this month. Congratulations!”

However, most primary care providers were concerned about the time and effort required to review the reports sent by online weight-loss programs. They thought it would add to their workload, so they preferred “if the website could give feedback to the physician that would not require a great effort on the physician’s part to access it.” Others thought that feedback from online weight-loss programs should arrive at a controllable frequency, so it would not be overwhelming. Lack of reimbursement was also mentioned as a factor. One provider was reluctant to review and respond to online weight-loss program reports because “currently the reimbursement structure, sad to say, doesn’t allow us to do this kind of work.” Another said, “Would I want, you know, fifty people telling me to look at their weight program per week and it’s not reimbursed? No.”

Most primary care providers would want to receive reports only when a patient was not meeting goals because “to get regular progress [reports] on patients who are doing okay is information overload.” If a patient is doing well, another participant said, “I don’t need all of the detail...it comes down to trying to figure out how much is really enough to trigger some action by us.” One participant stated:

...it probably would be beneficial to be able to track what they’re doing and then help them tweak things if we start to see they’re plateauing out on their weight or if they’re gaining weight instead of losing weight...

The primary care providers suggested ways to streamline the communication with online weight-loss programs. The first was to allow the providers to specify the frequency of such reports: “I actually prefer where I’m in control of how often I want to be updated.” The second was that online weight-loss programs provide reports electronically and integrate them into existing electronic health records. Providers also had other suggestions to make the online weight-loss program more accessible, such as offering them on mobile devices, on computers located in the clinic, and in other languages (eg, Spanish).

## Discussion

Structured online weight-loss programs promote modest weight loss among volunteers from nonclinical settings [6-9] and patients in the primary care setting [10,11]. By exploring the perspectives of primary care providers, we identified core issues to address in translating online weight-loss programs from research settings into routine primary care. This analysis revealed that many primary care providers are not incorporating online weight-loss programs in their patient care. However, the providers raised critical insights about the need for data on program effectiveness and safety; characteristics of patients most likely (in their view) to use online weight-loss programs; program features that providers are likely to endorse; and the types of feedback reports that would facilitate the integration of online weight-loss programs within primary care medicine.

The study had notable strengths. To our knowledge, this is the first qualitative study of clinicians’ perspectives on online weight-loss programs. Another strength was the inclusion of primary care providers from multiple practice settings, specialties, and professional designations (physicians and mid-level providers).

The study also had important limitations. The participants were mostly non-Hispanic white or Asian, from urban or suburban practices. Perspectives of providers from other ethnoracial backgrounds and rural settings might have yielded a more complete portrait of the topic. The study did not address the views of other stakeholders, such as patients, office staff, or designers and administrators of online weight-loss programs. Another limitation is that the questions presented in the focus group discussions were not constructed based on a specific theory, nor were they pilot tested before use. We constructed the questions to address clinically relevant gaps in knowledge.

Our results extend knowledge of clinicians’ views on referring patients to weight-loss or related resources. A need for better access to such resources has been demonstrated: while 79% of family medicine physicians in New Jersey thought it would be “very helpful” or “crucial” to have a list of community weight-loss resources, only 19% reported knowing “much” or “very much” about community resources for severely obese patients [18]. Likewise, in a national physician survey about diabetes self-management education (DSME) programs, primary care providers noted concerns such as “Do not have enough DSME referral sources” (45%), “Patients are told to do things I do not want” (44%), and “DSME programs do not have quality I want” (31%) [19]. Primary care providers in the current study expressed similar concerns about online weight-loss programs.

According to our study participants, an ideal online weight-loss program would provide a structured curriculum, goal-setting assistance, self-monitoring tools with customized feedback, peer support monitored by experts, and reports for primary care providers. Except for reports for clinicians, these features are common elements of online weight-loss programs [22,23]. Randomized trials have demonstrated the efficacy of such online programs for weight loss[6-9,12,24] as well as maintenance of weight loss [25]. However, the primary care providers in this study did not express awareness of research results or the actual online weight-loss programs used in the trials. This highlights the importance of bolstering efforts to disseminate research and translate interventions into the primary care setting [10,11].

The feasibility and impact of monitoring peer interactions are comparatively less clear. Online peer support holds promise as a useful resource for weight control [14-16], but primary care providers in our study were concerned that some weight-loss advice from online peers would be inaccurate. However, weight-loss advice on online forums has been found to be generally accurate, with medication-related advice more likely than other advice to be inaccurate [26]. Likewise, primary care providers and online forum users were comparable with respect to knowledge about an over-the-counter weight-loss medication, although knowledge in both groups was suboptimal [27]. The effect of expert forum monitoring on weight-loss outcomes remains to be determined.

Primary care providers also preferred that patients have access to online weight-loss programs at no cost. In a prior study, the introduction of out-of-pocket costs for patients reduced participation in and physician referrals to weight-loss and smoking-cessation programs [28]. If patients don’t pay, other sources of funding might include insurance carriers, employers, or advertising revenue (content of ads notwithstanding). Automating the counseling would presumably reduce costs. Automated online counseling and human email counseling were both superior to no counseling for weight loss at 3 months [9]. However, wholly automated obesity counseling has been found to be less effective than automated advice augmented with human behavioral email counseling [7]. Regardless of the strategies used to decrease cost, providing effective online weight-loss services at minimal or no cost to patients will require a collaborative effort among multiple stakeholders.

Primary care providers had mixed attitudes about receiving reports from online weight-loss programs, with the desire to track their patients’ progress balanced by concerns about time demands. Traditional ancillary providers (eg, physical therapists) send progress reports to referring clinicians. Reports from online weight-loss programs may be necessary if insurance companies were to cover the costs of accessing the programs. However, our results clearly indicate the importance of streamlining the process to minimize the burden on providers in reviewing the reports.

Our findings provide an in-depth view of primary care providers’ perspectives on integrating online weight-loss programs into routine clinical care, revealing important areas for research and development as online weight-loss programs continue to be evaluated in clinical populations. The study suggests that efforts are needed to test the feasibility and impact of expert monitoring of peer support forums, develop methods of sending reports to primary care providers about their patients’ progress that are acceptable to providers, and minimize costs to patients while providing structured behavioral support. Addressing concerns voiced by primary care providers will hopefully lead to sustainable partnerships with online weight-loss programs, with the end goal of providing patients with comprehensive weight management services.

## Abbreviations

DSME: diabetes self-management education
